# Ultrasound-Guided Axillary Access Using a Micropuncture Needle Versus Conventional Cephalic Venous Access for Implantation of Cardiac Devices: A Single-Center Randomized Trial

**DOI:** 10.3390/jpm14111084

**Published:** 2024-10-31

**Authors:** Georgios Leventopoulos, Christoforos K. Travlos, Athinagoras Theofilatos, Panagiota Spyropoulou, Angeliki Papageorgiou, Angelos Perperis, Rafail Koros, Athanasios Moulias, Ioanna Koniari, Periklis Davlouros

**Affiliations:** 1 Department of Cardiology, University Hospital of Patras, Rio, 26504 Patras, Greece; athinag@gmail.com (A.T.); pennyspyop@med.uoa.gr (P.S.); med5140@ac.upatras.gr (A.P.); angperperis@upatras.gr (A.P.); korosraf@hotmail.com (R.K.); amoulias@upatras.gr (A.M.); ioanna.koniari@nhs.net (I.K.); pdav@upatras.gr (P.D.); 2 Research Institute, McGill University Health Center, Montreal, QC H4A 3J1, Canada; christoforos.travlos@mail.mcgill.ca

**Keywords:** cardiac devices, venous access, ultrasound-guided, axillary access, micropuncture

## Abstract

(1) Background: Ultrasound-guided axillary (USAX) vein puncture is a relatively new method to obtain venous access for the implantation of cardiac implantable electronic devices (CIED). However, its use is limited as most of the operators are not familiar with this technique. Our aim was to investigate the safety and efficacy of the USAX compared with the traditional cephalic vein dissection for venous access in CIED implantation. (2) Methods: This was a single-center, randomized, controlled, superiority trial. A total of 114 patients were randomized (1:1 ratio) to either USAX (u/s axillary group; 59 patients) or cephalic vein access (cephalic group; 55 patients). The primary study endpoint was defined as successful placement of all leads via the chosen access. Secondary study endpoints included time from local anesthetic injection to lead advancement in the SVC, total procedure time (skin to skin), procedure-related complications and pain perception. (3) Results: USAX was superior to cephalic access in terms of primary endpoint (OR: 4.3, 95% CI: 1.3, 14.0; *p* = 0.012). Total procedure duration was higher in the cephalic group (55.15 ± 16.62 vs. 48.35 ± 12.81 min, *p* = 0.017) but there was neither a significant difference in fluoroscopy time (*p* = 0.872) nor in total radiation dose (*p* = 0.815). The level of pain was higher in the cephalic group (*p* = 0.016), while the rates of complications were similar in both groups (*p* > 0.05). (4) Conclusion: USAX was superior to cephalic access regarding success rate, total procedure duration and level of pain, while having no difference in complication rates.

## 1. Introduction

Cardiac implantable electronic devices (CIEDs) comprise the standard-of-care method for many potentially life-threatening conditions, including atrioventricular block or ventricular tachyarrhythmias. According to a survey conducted by the European Heart Rhythm Association, blind subclavian puncture and cephalic vein dissection are the most frequently adopted techniques regarding implantation of CIED leads in European medical facilities [1].

The subclavian vein approach has high success rates and allows the insertion of multiple leads, but it still includes central venous catheterization, which is associated with uncommon but potentially lethal complications [1,2] as well as higher long-term lead failure due to subclavian crush syndrome [3,4,5,6,7]. On the contrary, cephalic vein dissection is considered safer since central venous puncture is not required, with the downside of lower success rates and longer procedural duration [8]. Moreover, lead insertion and advancement heavily rely on vessel anatomy, tortuosity, diameter and operator’s skills [9], which culminate in success rates from 30% to 90% [10,11,12,13].

Another relatively new technique gaining popularity is the axillary vein puncture guided by ultrasound. The extrathoracic location of this vein helps to avoid complications associated with central venous puncture. In addition, its large caliper allows the insertion of multiple leads [7]. Ultrasound guidance provides significant advantages to this technique, since it allows direct visualization of the vasculature and the surrounding tissues. Therefore, access becomes safer and less time-consuming [14,15]. Among the strengths attributed to ultrasound-guided puncture are the faster learning curve, [14] the evaluation of venous patency before pocket creation, the particular appropriateness in cases of pre-existing leads [16,17] and the need of fewer venograms and less fluoroscopy compared to conventional techniques [18,19].

The current literature does not support the establishment of this method in everyday clinical practice with enough randomized trials. Our study aims to compare the safety and efficacy of ultrasound-guided axillary access using a micropuncture needle (USAX), with the traditional cephalic vein access performed by operators who were not self-trained.

## 2. Methods

### 2.1. Study Design

Our study is a single-center, randomized, controlled, superiority trial. In total, 114 patients were enrolled from May 2021 to October 2022 and were randomized by a computer algorithm in a 1:1 fashion to either USAX (investigational arm) or cephalic access (control arm). Operators were blinded about the type of access being used until the patient was on the table the day of the procedure.

### 2.2. Trial Population

All the patients who were referred for either single or dual chamber device implantation were included in the study. Patients received either a pacemaker (PM) or an implantable cardioverter defibrillator (ICD) according to their indication.

As inclusion criteria, all patients had to be over 18 years old with an indication of a single or dual chamber pacemaker/defibrillator after a signed consent form. Biventricular pacing, upgrading or revision procedures were excluded from randomization.

### 2.3. Procedure

All the procedures were performed at a tertiary center. Both operators (GL and AP) were electrophysiologists experienced in cephalic access (>400 cases). The prerequisite for starting patients’ recruitment was that the operators had undergone a training phase in USAX at the Interventional Radiology Laboratory and had completed at least 30 procedures. Abbott (Green Oaks, IL, USA) and Medtronic (Minneapolis, MN, USA) generators and leads were implanted. At each procedure, either GL or AP were first operators. Both urgent and elective cases were included in the study.

As preprocedural management, 1 gr of vancomycin was administered to each patient at least 2 h prior to the procedure. Regarding anticoagulation, if the patient was on apixaban or dabigatran, two prior doses were withheld, whereas with rivaroxaban, one was withheld. Patients on vitamin K antagonists should have an INR target less than 2.5 in order to proceed. Finally, the last dose of enoxaparin had to be administered at least 12 h before the procedure.

The type of access was disclosed to the operators (GL and AP) while the patient was prepped with chlorhexidine.

Left-sided implantation was preferred for both types of devices and was performed in 100% of cases. After the patient was prepped, 20 mL of lidocaine 1% was injected at the deltopectoral groove.

**Figure 1:**
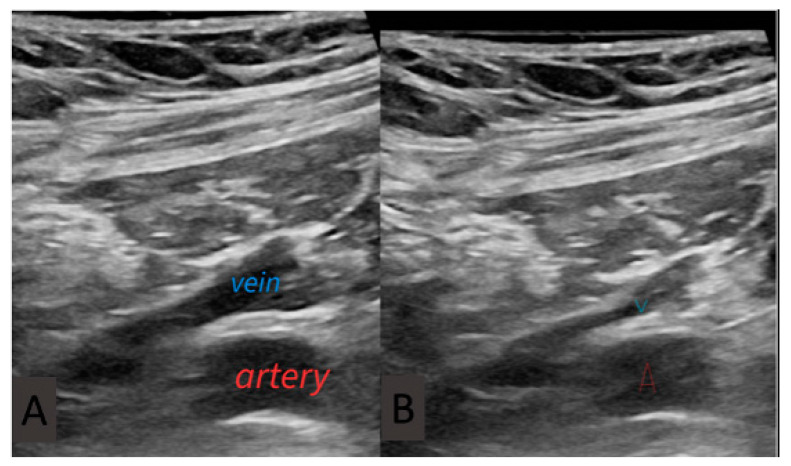
Long-axis view depicting axillary vein and artery. (**A**) Axillary vein is seen superior to the axillary artery. (**B**) Gentle pressure is applied through the probe, and the vein lumen collapses, confirming the fact that it is the vein and also confirming its patency. The arterial lumen remains unchanged under the pressure.

**Figure 2:**
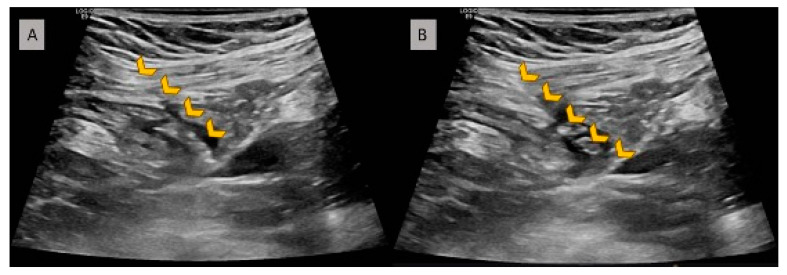
Images of ultrasound-guided axillary vein puncture (long-axis view—in-plane technique). The yellow markers indicate the needle. (**A**) Needle just before reaching the axillary vein. (**B**) The needle tip is inside the vessel lumen.

**Figure 3:**
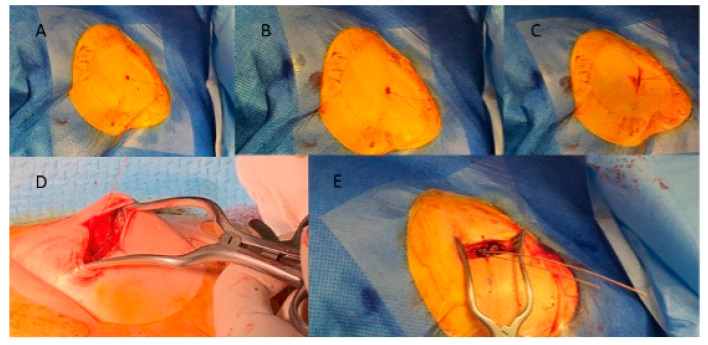
The wiring process after venous access. (**A**) A single 0.018′′ wire inside the vein, protruding through the skin. (**B**) The second 0.018′′ wire is placed following the second cannulation (in case of dual lead device). (**C**) Small incision made proximal (in this case caudally) to the inserted wires. (**D**) Tissues are dissected in the incision, and the wire is pulled through subcutaneously so that it now protrudes through the incision. (**E**) Both 0.018′′ wires are exchanged with 0.035′′ wires.

Axillary access: A portable handheld ultrasound scan (GE V scan extend with vascular linear probe 3.3–8.0 Hz) was covered by a sterile plastic sleeve and placed over the deltopectoral groove. Both axillary artery and vein were visualized in long- and short-axis planes (Figure 1A). Manual compression (Figure 1B) and color doppler were used to differentiate between artery and vein in case of uncertainty. Venous puncture was performed with a Micropuncture Kit (MAK 401 Merit Medical). A 21G needle attached to a 10 mL syringe was advanced either in plane or out of plane view (Figure 2). After venipuncture, a 0.018′′ guidewire was threaded through the needle. The course of the wire inside the axillary vein was confirmed with fluoroscopy. If the device had two leads, two separate micropuncture access points prior to the creation of the pocket were performed. Double-wiring was not allowed. Finally, two 0.018′′ wires were placed inside the vein (Figure 3B). As a next step, skin incision was performed cranially or caudally to the guidewire(s) and as close as possible to the guidewire entry site. After that, tissues below the skin incision were dissected, and the subcutaneous part of the guidewire was reached and pulled in such a way so that the distal part exited through the skin incision and not through the initial puncture site (Figure 3D). A 4F coaxial introducer was advanced through the 0.018′′ guidewire which was exchanged with a 0.035′′ guidewire in order to advance over the wire a conventional peel-away sheath. The lead was advanced through the sheath as per usual.

Cephalic: The cephalic vein was prepared, and with the cut-down technique, the lead was either advanced directly or with the use of a 0.035′′ guidewire. On this occasion, the lead was advanced into the vein through an introducer sheath. In case of failure, a hydrophilic 0.035′′ guidewire (Terumo) was used to facilitate wire advancement.

When the lead(s) was advanced at the superior vena cava (SVC), then the procedure was common for both arms. Active fixation leads were used in all patients. Atrial leads were placed at the right atrial appendage and ventricular leads at the mid-ventricular septum. Fluoroscopy was provided by a C arm (Ziehm Vision RFD Hybrid Edition) set at 4 frames per second with the appropriate collimation.

Anticoagulation was restarted 24–36 h after the procedure according to the operator’s discretion and the existence of hematomas. Bridging with enoxaparin was forbidden.

### 2.4. Study Endpoints

The primary study endpoint was defined as successful placement of all leads via the chosen access. In case of failure to place all leads via the chosen access, the following options were available:

- Crossover options for the USAX were in steps: (1) cephalic vein, (2) axillary vein with fluoroscopy with or without contrast venography and (3) subclavian vein. It must be clarified—as stated above—that double-wiring was not allowed. Therefore, if the second lead could not be advanced, this was considered a failure, and a crossover approach had to be followed.
- Crossover options for the cephalic vein arm were in steps: (1) axillary vein with fluoroscopy with or without venography and (2) subclavian vein.

Secondary study endpoints were time from local anesthetic injection to lead advancement in the SVC; total procedure time (skin to skin); and procedure-related complications, such as pneumothorax, cardiac tamponade, hematoma, lead dislodgement, patient’s hospital stay duration, fever, antibiotic treatment escalation and pain perception [use of visual analogue pain scale (VAPS) 0–10].

No time limit was set for the operator for both accesses. Regarding the USAX, no limit of puncture attempts was imposed on the operators, and puncture was performed before skin incision. One attempt was defined as a skin puncture and needle advancement till venous puncture without changing needle direction.

Hematomas were classified as clinically significant if the required evacuation resulted in prolongation of hospital stay and/or interruption of anticoagulation as defined in the Bruise 2 study [20]. Finally, it should be highlighted that according to our hospital’s policy, the minimum hospital stay after a device implantation was 2 days.

### 2.5. Statistical Analysis

Our primary hypothesis was that USAX would be superior to the cephalic access regarding the primary endpoint. All the endpoints were tested following the intention-to-treat principle. Based on existing data [21,22,23] and pilot data from our center, we considered for the sample size calculation a success rate of 65% in the cephalic group and 90% in the u/s axillary group. We calculated that 57 patients were needed in each group in order to have 90% statistical power and a two-sided a = 0.05.

The normality of the continuous outcomes distribution was tested using the Shapiro–Wilk test. Categorical variables are presented using absolute and relative frequencies (n, n%), while continuous variables are reported using mean values and standard deviations (mean ± SD) or median and interquartile range [median (Q1–Q3)] for skewed data. Outcomes between the two groups were compared using the Pearson’s chi square test or Fisher’s exact test, as deemed appropriate, for categorical variables. Student’s t test and the Mann–Whitney U test were used, as appropriate, for the investigation of an association between a continuous and a categorical variable. A multiple logistic regression model was used to adjust for confounding variables regarding our primary outcome and to explore the potential association of these variables with its occurrence. The independent variables fitted in the model were the implantation technique, age, sex, BMI, presence of heart failure, chronic kidney disease, chronic obstructive pulmonary disease, use of anticoagulation and type of device. The dependent variable was the successful placement of all leads. A stepwise forward method was employed to fit the independent variables in the model. Subgroup analyses regarding the primary endpoint were performed in different patients’ strata. Data analysis was performed with IBM SPSS 25.0 software (Statistical Package for Social Sciences), and two-sided *p*-values < 0.05 were considered to be statistically significant.

## 3. Results

### 3.1. Study Population

A total of 114 patients were randomized to either the cephalic vein access (cephalic group; 55 patients) or the USAX (u/s axillary group; 59 patients). Patients’ baseline and clinical characteristics were well balanced between the two groups and are shown in Table 1. The median age of the patients was 76 (68–83) years, 75.4% were male, and the median BMI was 27 (23.5–29). Half of the patients (45%) were under anticoagulation therapy, while the majority (65%) were referred for PM implantation. Hydrophilic wires were used in 13 patients in the cephalic group (24%).

### 3.2. Primary Endpoint

The primary endpoint was achieved in 55 patients (93.2%) in the u/s axillary group compared with 42 patients (76.4%) in the cephalic group (OR: 4.3, 95% CI: 1.3, 14.0; *p* = 0.012).

### 3.3. Secondary Endpoints

Regarding the secondary endpoints, total procedure duration was significantly higher in the cephalic group compared with the u/s axillary group (55.15 ± 16.62 vs. 48.35 ± 12.81 min, *p* = 0.017). Also significantly higher was the time from local anesthetic injection to lead advancement in the SVC in the cephalic group (15.5 min, 11.2–24.5) compared with the axillary (12 min, 9–15; *p* = 0.001). However, there was neither a significant difference in the fluoroscopy time (cephalic group; 188.76 ± 140.78 s vs. u/s axillary group; 194.30 ± 209.96 s, *p* = 0.872) nor in the total radiation dose between the two groups (u/s axillary: 167 DAP (cGy/cm^2^), 106–398 vs. cephalic: 182 DAP (cGy/cm^2^), 109–360: *p* = 0.815). The level of pain was found to be significantly higher in the cephalic than the u/s axillary group (3.88 ± 2.37 vs. 2.69 ± 2.57 in VAPS, *p* = 0.016), but there was no difference in the duration of hospitalization between the two groups (cephalic group; 3.83 ± 5.76 days vs. u/s axillary group; 2.46 ± 1.55 days, *p* = 0.091). All the results are summarized in Table 2.

### 3.4. Secondary Analyses of the Primary Outcome

In subgroup analysis, it was shown that in patients with a BMI ≥ 25 (75 patients), there was a significantly higher occurrence of the primary outcome in the u/s axillary group (94.9%) versus the cephalic group (75.0%; OR: 6.33, 95% CI: 2.31, 17.33; *p* < 0.001), but in patients with a BMI < 25 (28 patients), the occurrence of the primary outcome was the same in both groups (93.3 vs. 92.3%; OR: 1.16, 95% CI: 0.40, 3.32; *p* > 0.05). The occurrence of the primary outcome was significantly higher in the u/s axillary group both in patients with (71.4% vs. 55.6; OR: 1.92, 95% CI: 1.07, 3.46 *p* < 0.05) and without chronic kidney disease (CKD; 96% vs. 82.2%; OR: 5.27, 95% CI: 1.71, 16.19; *p* < 0.05). However, when patients were divided according to the number of leads placed (ventricular or atrial + ventricular lead) or according to the type of implanted device (PM or ICD), the occurrence of the primary outcome had no significant difference between the two groups (*p* > 0.05).

In a multivariable regression analysis, the u/s axillary access (OR 8.82; CI 95%: 1.26, 61.85) and the presence of CKD (OR 0.047; CI 95%: 0.01, 0.35) were the only independent variables that could predict the primary outcome.

### 3.5. Lead Insertion Failures

Thirteen failures occurred in the cephalic group (24%) and four in the u/s axillary group (7%). In the cephalic group, there were ten crossovers to axillary vein access with fluoroscopy without using contrast venography, one crossover to axillary vein access with fluoroscopy using contrast venography and two crossovers to subclavian vein access. In the u/s axillary group, there were three crossovers to axillary vein access with fluoroscopy without using contrast venography and one crossover to subclavian vein access. The causes of failure in the cephalic group were inability to locate the vein (n = 3, 5.5%), small diameter of the cephalic vein (n = 4, 7.3%), cephalic vein injury (n = 3, 5.5%) and vein spasm (n = 3, 5.5%). In the u/s axillary group, the causes of failure were inability to puncture (n = 2, 3.4%) and inability to insert wire (n = 2, 3.4%).

### 3.6. Complications

Procedural complications included the presence of significant and non-significant hematoma, cardiac tamponade, pneumothorax, fever, lead dislodgement and prolonged use of antibiotics after the procedure. There was no significant difference in each type of complication between the two groups (*p* > 0.05). The percentages of each complication by group can be found in Table 3.

More specifically with regard to the presence of hematomas, we found that there was no significant relation between the appearance of hematomas and the implantation technique (*p* = 1.000) or the type of the device (*p* = 0.515) used. Nevertheless, hematomas occurred more frequently in patients on anticoagulation compared to patients who did not receive anticoagulants (35.3% vs. 17.9%, *p* = 0.040).

Analyzing the potential factors that could contribute to the size of hematomas, such as renal function, pre-procedural use of enoxaparin and time since last dose of anticoagulant, we found that there was not a statistically significant association between any of them and the size of the hematoma (*p* > 0.050).

## 4. Discussion

Our randomized study compared USAX using micropuncture needle with conventional cephalic vein cutdown in CEIDs, and it is, to our knowledge, the second largest so far (114 patients). It is, however, the largest randomized trial in terms of conventional percutaneous USAX with the use of a micropuncture needle (21 g) and the performance of separate micropuncture accesses—if needed—prior to the creation of the pocket. Our main findings are the superiority of USAX compared with cephalic vein in terms of success rate, which was our primary endpoint. Regarding the secondary endpoints, USAX was related to statistically significant (i) lower total procedure time, (ii) lower time to place lead(s) at the SVC and (iii) lower pain assessment, while there was no difference in complications, days of hospitalization, fluoroscopy time and radiation exposure.

According to the literature, the US-guided approach has been examined in several studies [18,19,21,22,24,25,26,27,28,29]. We report a success in USAX of 93.1%, with a mean number of punctures of 1.15. Our data are in line with to-date literature, in which a success rate ranging between 88% and 99% is reported [18,19,21,22,24,25,26,27,28,29].

In addition, several non-randomized trials present exceptionally high success rates for the ultrasound-guided axillary technique, even reaching 99% [27,28,29], and quite recently the largest to-date RCT comparing USAX vs. cephalic was published and reported a 99% success rate (which would have been 100% had it not been for a case of persistent left SVC) [21]. Esmaiel et al., [28] Jones et al. [15] and the investigators of the ACCESS trial [24] formed the device pocket and proceeded with the venipuncture through the initial incision. The small size of the incision most of the times renders the contact between the linear probe used in our study and the tissues suboptimal, thus providing lesser image quality and vessel visualization due to air interference. This problem was solved in the ACCESS trial [24], where a special “hockey stick”-shaped probe was used, skyrocketing the success rate to 99% versus the 88% success rate of Jones et al. [15], who used a classic linear probe during intrapocket ultrasound-guided venipuncture of the axillary vein. However, this is novel special equipment not commonly found in an electrophysiology laboratory. Esmaiel et al. [28] also used a micro-convex probe and reported success rates of 99% with intrapocket axillary puncture. The aforementioned studies suggest that, when a dedicated probe is used, intrapocket ultrasound-guided axillary access is an intriguing option, especially in patients with deeper veins or increased BMI. As the equipment described above was not available in our study, ultrasound-guided intrapocket axillary venipuncture was not included in the crossover options of cephalic failure.

Cephalic vein access was successful in 76% of our patients (42/55). This result is in line with the recent literature, including several studies when no time limit was applied [14,22,30]. The reasons for failure are presented in the Results section. Of note, Jones et al. [15] reports a higher success rate (87%) for cephalic access in a non-randomized study. In the study by Tagliari et al. [22], one of the operators was a cardiothoracic surgeon, who are known to be more familiar with the cephalic cutdown technique. We have to underline that in our study all procedures were performed by electrophysiologists without the participation of a cardiothoracic surgeon, although both our operators were very experienced.

The first RCT comparing percutaneous ultrasound-guided axillary access with conventional cephalic vein access was conducted in 2020 by Tagliari et al. and included 88 patients [22]. They reported a significantly higher success rate in the axillary group (97.7%) compared to the cephalic group (54.5%) and fewer complications at 30-day follow-up (2.3% U/S axillary vs. 11.4% cephalic). However, it has to be noted that most of the minor complications (6.8%) were lead displacements that might have been unrelated to the access choice. Unsuccessful access was also defined as access not obtained after three punctures or after 15 min of trying. Such limitations were not applied in our study. Their results (97.7% vs. 79%) are similar to ours (93.1% vs. 76.4%) in terms of success rates when no time limit was applied. However, the abovementioned study [22] demonstrated a shorter total procedure time in its axillary arm compared to ours (40 min vs. 48 min). We assume this can be attributed to the following reasons: (a) the micropuncture technique with 21 g needle requires a two-step approach with advancement of a 0.018′′ guidewire that is later exchanged for a 0.035′′ one; (b) regarding dual lead devices, each lead required a separate access (no double-wiring as in Tagliari et al.); and (c) more than half (57%) of the procedures were performed by a single operator in our cohort. As mentioned by Chandler et al., [18] total procedure time is reduced with the participation of the second operator/fellow (12 min vs. 6 min, *p* < 0.01 in their study).

No statistically significant difference was found in fluoroscopy time and radiation exposure between the two groups in our study. Our fluoroscopy time in the US-guided group was 194 s vs. 188 in the cephalic group. A similar fluoroscopy time was reported by Courtney et al. [25] (192 s) and Chandler et al. [18] (216 s) in the USAX arm.

On the other hand, the total procedure time was significantly shorter in the US-guided axillary group compared to the cephalic (48 min vs. 55 min). This is easily explained by the higher crossover rate of cephalic access. Moreover, there was no time limit for the cannulation of the cephalic vein before the operator decided to abandon this approach.

Regarding the safety aspect, the complication rate was similar in both. In the U/S-guided axillary group, there was one pneumothorax noted. However, this was treated conservatively as it was small. This may be the positive impact of using the fine 21 g micropuncture needle that may be forgiving even in cases of accidental pleural puncture. Although the proportion of patients receiving anticoagulants was similar in both groups, it was noticed that cephalic access was related to larger-sized non-significant hematomas. This could explain, in turn, the numerically higher incidence of fever and the higher need for antibiotics and longer (in days) hospital stays in the cephalic group. Although not statistically significant, there was a trend towards shorter hospital stays in the axillary arm group (2.46 days vs. 3.83 days, *p* = 0.09).

Finally, regarding predicting factors, it was found that a BMI ≥ 25 is related to a lower success rate in the cephalic group, whereas no difference in success rate was noted amongst patients with a BMI less than 25. This observation highlights the anatomical restrictions that are associated with the cephalic cutdown technique when it is located at a very deep plane. Additionally, chronic kidney disease is a predictor of failure for either the cephalic or axillary access. The emergence of GFR as an independent predictor of implantation failure can be explained by the fact that GFR itself is an indicator of frailty and harder venous access (higher tortuosity, calcification, previous use for dialysis).

### 4.1. Limitations

Our study has some limitations. First, it is a single-center randomized study involving two operators. The study design did not allow our operators to be blinded regarding the technique. Still, they were blinded to the group assignment. Second, cardiac resynchronization therapy (CRT) systems and device upgrades were not included in the randomization. Third, our study was designed to compare only the primary endpoint, which implies that the results of the secondary endpoints need to be further explored. Fourth, both operators—albeit not self-trained—were still less experienced in USAX than in cephalic access. Moreover, we acknowledge that this drawback cannot be offset by the initial training of 30 cases that each of the operators received from the radiologists. However, our aim was to present a pragmatic approach as it stands in the striking majority of electrophysiology labs worldwide, where most of the device operators are not trained in ultrasound-guided access and the mainstay ultrasound equipment is a vascular linear probe used for femoral access. Fifth, our study design did not include the follow-up of patients post-discharge. Apart from this, there was no close follow-up of our patients beyond their hospital stay in order to monitor lead complications related to the access.

### 4.2. Clinical Implications

Our point is that a linear probe—that is commonly used for femoral access—can also be used to facilitate axillary access in device procedures. Our results denote that the use of a micropuncture needle can be the first option. However, as a failure rate still exists, training on other modes of access—and especially the cephalic—should not be neglected and remain of paramount importance.

## 5. Conclusions

A strategy that includes the use of a micropuncture needle for device implantation is associated with a significantly higher success rate, less pain and shorter total procedure duration compared to a cephalic vein implantation strategy, without a difference in complication rates between the two strategies. The absence of CKD was independently associated with USAX superiority over the cephalic strategy. A high BMI (>25) was found to be related to significantly lower success rates in the cephalic group, and chronic kidney disease was an independent predictor of lower success in both groups.

## Figures and Tables

**Table 1:** Patients’ baseline characteristics.

	U/s Axillary Group (n = 59)	Cephalic Group (n = 55)	*p*-Value
Age—years	76 (66.50–81.50)	76 (70.75–83.25)	0.379
Male sex—n (%)	45 (76.3)	41 (74.5)	0.831
Body mass index—kg/m^2^	27 (23–29)	27 (24, 29)	0.870
Known coronary artery disease—n (%)	22 (37.3)	22 (40.0)	0.817
Prior MI—n (%)	15 (25.4)	16 (29.1)	0.697
Heart failure—n (%)	16 (27.1)	11 (20.0)	0.345
Atrial fibrillation—n (%)	44 (74.6)	40 (72.7)	0.832
Diabetes mellitus—n (%)	23 (38.9)	16 (29.1)	0.266
Hypertension—n (%)	40 (67.8)	43 (78.2)	0.252
Dyslipidemia—n (%)	30 (50.8)	34 (61.8)	0.271
COPD—n (%)	2 (3.4)	2 (3.6)	0.956
CKD—n (%)	7 (11.9)	9 (16.4)	0.511
***Concomitant medications***			
Anticoagulants—n (%)	26 (44.1)	25 (45.5)	0.943
Aspirin—n (%)	17 (28.8)	15 (27.3)	0.812
P2Y12—n (%)	16 (27.1)	13 (23.6)	0.632
***Type of Device***			
Pacemaker—n (%)	39 (66.1)	35 (63.6)	0.783
ICD—n (%)	20 (33.9)	20 (36.4)	
***Indication PPM***	(39)	(35)	
2nd degree AV block—n (%)	6 (15.4)	3 (8.6)	0.371
3rd degree AV block—n (%)	11 (28.2)	12 (34.2)	0.624
Sick sinus syndrome—n (%)	15 (38.5)	10 (28.6)	0.369
Slow AF	6 (15.4)	9 (25.7)	0.270
Syncope and bifascicular block	1 (2.6)	1 (2.9)	0.938

Data are presented as median (interquartile range) or number (%). MI: Myocardial infarction; COPD: chronic obstructive pulmonary disease; CKD: chronic kidney disease; ICD: implantable cardioverter defibrillator; AV: atrioventricular.

**Table 2:** Study endpoints.

	U/s Axillary Group (n = 59)	Cephalic Group (n = 55)	Odds Ratio (95% CI)	*p*-Value
**Primary endpoint**				
Successful placement of all leads—n (%)	55 (93.2)	42 (76.4)	4.3 (1.3–14.0)	**0.012**
**Secondary endpoints**				
Total procedure duration—min	48.35 ± 12.81	55.15 ± 16.62	-	**0.017**
Time to SVC—min	12 (9–15)	15.5 (11.2–24.5)	-	**0.001**
Fluoroscopy time—s	194.30 ± 209.96	188.76 ± 140.78	-	0.872
Total radiation dose—DAP (cGy/cm^2^)	167 (106–398)	182 (109–360)	-	0.815
Level of pain—VAS	2.69 ± 2.57	3.88 ± 2.37	-	**0.016**
Duration of hospitalization—days	2.46 ± 1.55	3.83 ± 5.76	-	0.091

Data are presented as mean ± standard deviation, median (interquartile range), OR (95%CI) or number (%). SVC: superior vena cava; VAS: visual analog scale.

**Table 3:** Complications.

	Cephalic Group (N = 55)	U/s Axillary Group (N = 59)
**MAJOR**		
Pneumothorax—n (%)	0	1 (1.7)
Tamponade—n (%)	1 (1.8)	0
Significant hematoma	2 (3.6)	1 (1.7)
**MINOR**		
Non-significant hematoma—n (%) Size of non-significant hematoma—cm	11 (20) 11 [9,12]	12 (20) 8.5 [3,12]
Fever—n (%)	7 (13)	2 (3.4)
Lead dislodgement—n (%)	0	1 (1.7)
Prolonged antibiotic intake post-operatively—n (%)	13 (24)	8 (14.5)

Data are presented as number (%).

## Data Availability

Data are available from the corresponding author upon reasonable request.
